# Phase I prospective trial of TAS-102 (trifluridine and tipiracil) and radioembolization with ^90^Y resin microspheres for chemo-refractory colorectal liver metastases

**DOI:** 10.1186/s12885-022-10401-0

**Published:** 2022-12-13

**Authors:** Nicholas Fidelman, Chloe E. Atreya, Madeline Griffith, M. Alexandra Milloy, Julia Carnevale, Pelin Cinar, Alan P. Venook, Katherine Van Loon

**Affiliations:** University of California, San Francisco, USA

**Keywords:** TAS-102, Lonsurf, Trifluridine, Tipiracil, Yttrium-90, Radioembolization, colon cancer

## Abstract

**Background:**

Extrahepatic disease progression limits clinical efficacy of Yttrium-90 (^90^Y) radioembolization (TARE) for patients with chemotherapy-refractory metastatic colorectal cancer (mCRC). Trifluridine and tipiracil (TAS-102) has overall survival benefit for patients with refractory mCRC and may be a radiosensitizer.

**Methods:**

Sequential lobar TARE using ^90^Y resin microspheres in combination with TAS-102 in 28-day cycles were used to treat adult patients with bilobar liver-dominant chemo-refractory mCRC according to 3 + 3 dose escalation design with a 12-patient dose expansion cohort. Study objectives were to establish safety and determine maximum tolerated dose (MTD) of TAS-102 in combination with TARE.

**Results:**

A total of 21 patients (14 women, 7 men) with median age of 60 years were enrolled. No dose limiting toxicities were observed. Treatment related severe adverse events included cytopenias (10 patients, 48%) and radioembolization-induced liver disease (2 patients, 10%). Disease control rate in the liver lobes treated with TARE was 100%. Best observed radiographic responses were partial response for 4 patients (19%) and stable disease for 12 patients (57%).

**Conclusions:**

The combination of TAS-102 and TARE for patients with liver-dominant mCRC is safe and consistently achieves disease control within the liver.

**Trial registration:**

ClinicalTrials.gov identifier NCT02602327 (first posted 11/11/2015).

**Supplementary Information:**

The online version contains supplementary material available at 10.1186/s12885-022-10401-0.

## Background

Liver is the most common site of metastatic involvement by colorectal cancer (mCRC), however, only a small proportion of patients are candidates for resection of hepatic metastases [1]. For patients with liver-dominant metastatic disease, hepatic decompensation contributes to morbidity, and liver failure is often the direct cause of death [1, 2]. Control of tumor progression in the liver may, therefore, improve patient outcomes for patients with mCRC with liver-dominant disease.

Loco-regional endovascular therapies, including selective internal radiation therapy (hereafter referred to as transarterial radioembolization, TARE), are based on the principle that tumors derive their blood supply from the hepatic artery, and tumor perfusion is several-fold higher than perfusion of the surrounding liver parenchyma [3]. Radioembolization involves trans-catheter arterial delivery of 20-60 mm microspheres containing Yttrium-90 (^90^Y) radioisotope into the tumor microvasculature [4].

The safety of TARE for colorectal liver metastases (CRLM) has been previously established [5], and prior studies have suggested that TARE may confer a progression free survival (PFS) advantage in patients with chemotherapy-refractory mCRC when compared to single agent 5-FU [5–8]. However, when used in the first-line setting, phase III data has failed to demonstrate improvement in PFS in chemotherapy-naïve patients with unresectable, liver only or liver dominant disease [9].

A prospective study that included 21 patients with chemotherapy-refractory mCRC who were treated with TARE [10] demonstrated a median PFS of 1.0 month (range 0.8–21.9 months). Median PFS in the treated liver lobe(s) was 4.5 months (range 1.0–9.8 months), whereas median untreated liver lobe and extrahepatic PFS was 1.0 month (ranges 1.0–24 months and 1.0–12.8 months, respectively). These results suggested that overall PFS was limited by extrahepatic disease progression. This finding highlighted a need for development of novel treatment approaches that combine ^90^Y radioembolization with effective systemic chemotherapy for patients with chemotherapy-refractory mCRC.

TAS-102 is an oral nucleoside antitumor agent that combines the drugs trifluridine and tipiracil hydrochloride. TAS-102 was approved by the United States Food and Drug Administration in 2015 for the treatment of refractory mCRC. This approval was based upon results of a phase 3 randomized controlled trial [11] that evaluated TAS-102 for patients with chemotherapy-refractory CRC and reported a median OS of 7.1 months (95% CI: 6.5–7.8) and 5.3 months (95% CI: 4.6–6.0) for TAS-102 and placebo, respectively. Median PFS, overall response rate (ORR), and disease control rate (DCR) for TAS-102 and placebo were 2.0 months vs. 1.7 months (NS), 1.6% vs. 0.4% (NS), and 44.0% vs. 16.3% (*p* < 0.0001), respectively. The most frequent grade 3 or higher AEs with TAS-102 or placebo (observed in at least 10% of patients for TAS-102) were neutropenia (34.9% for TAS-102, 0% for placebo), leukopenia (12.8, 0%), and anemia (16.5, 2.6%) [11]. Transaminitis and hyperbilirubinemia occurred more commonly in the placebo group (AST 4% for TAS-102, 6% for placebo; hyperbilirubinemia 9% for TAS-102, 12% for placebo). Because of its mechanism of action, TAS-102 also has radiosensitizer property [12].

Benefit of single agent TAS-102 against chemotherapy-refractory mCRC and the drug’s promise as a radiosensitizer made TAS-102 a potential candidate drug for testing in combination with TARE using ^90^Y resin microspheres in patients with liver-dominant chemotherapy-refractory mCRC. The choice of TAS-102 over 5-FU or its oral prodrug, capecitabine, in this patient population was dictated by the premise that continued use of a single agent 5-FU was not likely to be clinically beneficial for patients with mCRC who have demonstrated disease progression on multiple lines of 5-FU-based therapy.

## Methods

### Study design and conduct

**Fig. 1:**
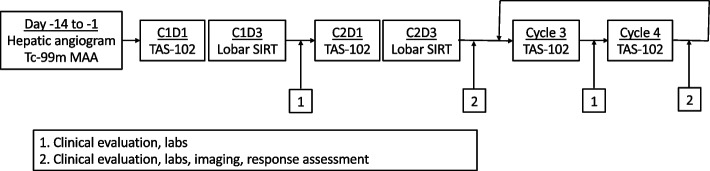
Graphic overview of the study design

This was a phase I dose escalation study (3 + 3 design) with a dose expansion arm (12 patients) designed to evaluate safety of the combination of TAS-102 and TARE using ^90^Y resin microspheres for patients with chemotherapy-refractory liver-dominant mCRC (ClinicalTrials.gov identifier: NCT02602327, first posted 11/11/2015). A graphic overview of the study is provided in Fig. 1. The study was approved by the Institutional Review Board at University of California San Francisco. The study was performed in accordance with the Declaration of Helsinki. All patients signed written informed consent after being advised of risks, benefits, and alternatives of study treatments and trial participation.

### Patient population

Key inclusion criteria were: 1) a diagnosis of progressive metastatic unresectable colon or rectal adenocarcinoma with liver dominant bilobar disease; 2) progression or intolerance to at least two prior lines of systemic therapy; 3) tumor replacement < 50% of total liver volume; 4) adequate hepatic laboratory parameters within 30 days of treatment, including normal serum bilirubin, alanine and/or aspartate aminotransferase < 5 times upper normal limit, albumin > 2.0 g/dL, and absence of clinically evident ascites; 5) adequate bone marrow reserve, including absolute neutrophil count (ANC) > 1500/ml and platelet count > 75,000/ml; and 5) Eastern Cooperative Oncology Group (ECOG) performance status 0 or 1. Key exclusion criteria were: 1) significant extrahepatic disease, defined as symptomatic extrahepatic disease (including primary tumor, if unresected), greater than 10 pulmonary nodules (each < 20 mm in diameter or combined diameter of all pulmonary nodules > 15 cm), and/or peritoneal carcinomatosis; 2) potential delivery of greater than 30Gy of radiation to the lungs during a single ^90^Y resin microsphere administration or cumulative delivery of greater than 50Gy to the lungs over multiple treatments; 3) evidence of any detectable flow to the stomach or duodenum mapped by Technetium-99 m macroaggregated albumin (Tc-99 m MAA), despite embolization aimed to stop such flow; 4) previous radiation therapy to the lungs and/or to the upper abdomen; 5) receipt of chemotherapy within 14 days prior to study treatment; and 6) history of biliary tract instrumentation. Of note, due to the possible increase in hepatotoxicity associated with whole liver single session TARE [13], sequential lobar TARE treatments were performed during the study.

Eligible patients were identified and referred by a medical oncologist (CEA, JC, PC, KVL, or APV). All study treatments were performed between February 2017 and December 2021. Hepatic disease progression with liver-dominant growth pattern was established by cross-sectional imaging (CT or MRI) obtained within 30 days of initiation of study treatment. A clinical evaluation including medical history, physical examination, Eastern Cooperative Oncology Group (ECOG) performance status, and laboratory tests (complete blood count [CBC], metabolic and coagulation panels, as well as carcinoembryonic antigen [CEA]) was performed within approximately 30 days prior to the initiation of study treatment. Between 5 and 14 days prior to the first TARE, patients underwent a planning visceral arteriography, protective coil embolization of hepatic artery branch vessels, and a lung shunt study using 1.0–1.8 mCi of Tc-99 m MAA particles. Lung shunt fraction (LSF) was calculated on the basis of planar images using standard methodology [14].

### TAS-102 administration

Standard 3 + 3 design was used for the dose escalation phase. TAS-102 (Lonsurf®, Taiho Pharmaceutical, Princeton NJ, USA) doses were 20 mg/m^2^ (cohort 1), 27.5 mg/m^2^ (cohort 2), and 35 mg/m^2^ up to a maximum of 80 mg (cohort 3) twice per day on days 1–5 and 8–12 of the first two 28-day cycles. Patients were provided with paper calendars for tracking of doses taken. Morning dose of TAS-102 was not administered on C1D3 and C2D3 (radioembolization days) due to patients’ fasting status. During cycles 1 and 2 only, last dose of TAS-102 was in the morning on C1D13 and C2D13. No dose reductions were permitted during cycle 1. Delay of cycle 2 up to 28 days was allowed in the setting of ANC < 1500/μl, platelet count < 75,000/μl, AST > 10X upper normal limit, ALT >10X upper normal limit, serum bilirubin > 2.0 mg/dl, serum creatinine > 2.0 mg/dl, and/or ECOG PS > 2. Patients who required a dose delay were evaluated weekly. Cycle 2 was started upon improvement of laboratory value abnormalities to Common Terminology Criteria for Adverse Events (CTCAE) grade 1 or better, and/or ECOG PS improvement to 0 or 1. For patients assigned to dose escalation cohorts 2 and 3, and dose expansion cohort, who required a dose delay, TAS-102 dose was reduced to 20 mg/m^2^ (cohort 2) or 27 mg/m^2^ (cohort 3 and dose expansion cohort). The lowest therapeutic dose of TAS-102 was assumed to be 20 mg/m^2^ twice per day. Therefore, dose reduction below 20 mg/m^2^ was not permitted.

Only patients with bilobar disease distribution were included in the study in order to adhere to a consistent dose limiting toxicity (DLT) window of 56 days, which reflected two back-to-back 28-day cycles of TAS-102 in combination with TARE. DLT parameters were ANC < 500/μl, platelet count < 50,000/μl, serum bilirubin > 3.9 mg/dl, AST > 20X upper normal limit, ALT >20X upper normal limit, febrile neutropenia, CTCAE grade 3 ascites, and administration of < 75% of the intended dose of TAS-102.

Maximum tolerated dose of TAS-102 was not reached during the dose escalation phase. For the dose expansion phase, patients received TAS-102 at the dose of 35 mg/m^2^ up to a maximum of 80 mg twice per day. Similar to the dose escalation phase, morning dose of TAS-102 was not administered on C1D3 and C2D3 (TARE days) due to patients’ fasting status. During cycles 1 and 2 only, last dose of TAS-102 was in the morning on C1D13 and C2D13.

Beginning with cycle 3, TAS-102 was administered at 35 mg/m^2^ per day in two divided doses (up to a maximum of 80 mg per dose) on days 1–5 and 8–12 of each 28-day cycle until 1) radiographic progression (Response Evaluation Criteria in Solid Tumors [RECIST] version 1.1); and/or 2) development of dose limiting toxicity; and/or 3) sustained functional decline to ECOG PS > 2 lasting more than 4 weeks. Dose delay guidelines were the same as for cycles 1 and 2. After dose delay, TAS-102 was restarted at a reduced dose level (dose level − 1: 30 mg/m^2^; dose level − 2: 25 mg/m^2^; dose level − 3: 20 mg/m^2^). Rechallenge at the full dose of TAS-102 (35 mg/m^2^) was allowed beginning with cycle 5. However, if dose reduction during cycle 5 or a subsequent cycle was required, repeat dose reescalation was not permitted. Growth factor support was allowed beginning with cycle 3.

### ^90^Y radioembolization and dosimetry

Lobar TARE was performed using ^90^Y resin microspheres (SIRTEX Medical, Ltd., Woburn, MA, USA) for all patients on day 3 of cycles 1 and 2 using standard methodology and administration technique [14]. The liver lobe harboring the highest volume of metastatic disease was treated first. Required ^90^Y resin microsphere activity was determined based on the body surface area (BSA) method. Expected absorbed lung and liver doses were calculated using the Medical Internal Radiation Dose (MIRD) model [14].

All TARE procedures were performed by one interventional radiologist with 10 years of experience (NF). A manufacturer-supplied administration set was used for delivery of ^90^Y resin microspheres. Patients were admitted to the hospital overnight for observation and were discharged on the following day. After each radioembolization procedure, patients were instructed to take a proton pump inhibitor (PPI) for 30 days for gastrointestinal ulcer prophylaxis, as well as methylprednisolone and ursodiol for 60 days for prevention of radiation-induced liver disease [13]. Discharge prescriptions also included a broad-spectrum antibiotic for 7 days (moxifloxacin, amoxicillin, or clindamycin), as well as an opiate analgesic, and an antiemetic on as needed basis.

### Follow-up evaluation

Follow-up clinical evaluations (interval history and physical examination, CBC with differential and serum chemistries including liver function tests and albumin) were performed approximately 14 days after starting cycles 1 and 2, and approximately 30 days after starting every treatment cycle. Imaging follow-up with a contrast-enhanced CT or MRI of the abdomen and pelvis as well as a chest CT was obtained approximately 30 days after the start of every even-numbered treatment cycle. Additional anti-tumor therapies were prescribed (when appropriate) at least 30 days after completion of study treatment.

### Criteria for evaluation

Primary objectives were to determine 1) maximum tolerated dose (MTD) of TAS-102 when used in combination with liver radioembolization using ^90^Y resin microspheres; 2) dose limiting toxicities; and 3) safety (adverse events) of TAS-102/^90^Y radioembolization combination therapy. Secondary objectives were 1) best observed radiographic response rate (by RECIST version 1.1); 2) progression-free survival (overall, hepatic, extrahepatic); 3) overall survival. Standard definitions of treatment efficacy parameters were used [15].

### Statistical analysis

Data were tabulated and analyzed using Excel (Microsoft, Redmond WA, USA). Survival data were reported using Kaplan-Meier method. Duration of hepatic and extrahepatic PFS was compared using the log rank test. Data were censored on August 1, 2022.

## Results

### Patient characteristics

Between February 2017 and February 2021, 26 patients were referred for study participation. Two patients were excluded due to inadequate liver function, another two patients were excluded due to lack of bilobar liver disease, and one patient was not enrolled due to excessive lung disease burden. A total of 21 adult patients with liver-dominant bilobar colorectal cancer metastases were enrolled. Median age at enrollment was 60 years (range 33–75 years), and 14 (67%) patients were women (Table 1). An asymptomatic primary tumor was in place in 13 patients (62%), while 14 patients (67%) had asymptomatic low-volume extrahepatic metastatic disease to the lungs (*n* = 8), lymph nodes (*n* = 5), and adrenal gland (*n* = 1) at the time of enrollment. Ten patients (48%) had less than 25% of liver parenchyma replaced by tumor, while 11 patients demonstrated 26–50% replacement of normal liver parenchyma. All patients had bilobar hepatic metastases.

**Table 1:** Baseline clinical and laboratory findings

Clinical parameter	*N* = 21
Age, years, median (range)	60 (33–75)
Gender	
Male, n (%)	7 (33)
Female, n (%)	14 (67)
Tumor origin	
Colon, n (%)	19 (90)
Rectum, n (%)	2 (10)
Primary in place, n (%)	13 (62)
Extrahepatic disease, n (%)	14 (67)
Extrahepatic disease sites	
Lungs	8
Lymph nodes	5
Peritoneum	1
Adrenal gland	1
Prior systemic therapy	
5-FU, oxaliplatin, irinotecan, and bevacizumab	21 (100%)
EGFR inhibitor	9 (43)
Regorafenib	4 (19)
ECOG PS	
0 / 1	6 / 15
Liver replacement, n (%)	
< 25%	10 (48)
26–50%	11 (52)

*Abbreviations: 5-FU* 5-fluorouracil, *ECOG PS* Eastern Cooperative Oncology Group Performance Status

All patients had demonstrated disease progression on and/or intolerance to fluoropyrimidines, oxaliplatin, irinotecan, and bevacizumab. Of the 21 participants, nine (43%) were previously treated with an epidermal growth factor receptor (EGFR) inhibitor, and four (19%) had received regorafenib prior to trial enrollment. Five patients (24%) had previously undergone wedge resection of liver metastases with or without concomitant thermal ablation. None of the patients had received any other liver-directed therapies including trans-catheter therapies, percutaneous ablation, or external beam radiation therapy prior to enrollment. All 21 patients have completed protocol-mandated follow-up and were deemed evaluable for toxicity and response assessment.

### TAS-102 administration

Among study participants, a median of four cycles (range 1–10 cycles) of TAS-102 were administered per patient. Dose delays were required for 11 (52%) patients. Delay lengths were either one week (seven patients) or two weeks (six patients) in duration. A total of 11 patients (52%) needed reductions of TAS-102 doses most commonly due to neutropenia and thrombocytopenia. Most of the dose reductions were from 35 mg/m^2^ to 30 mg/m^2^ with cycle 4 (9 patients) and from 30 mg/m^2^ to 25 mg/m^2^ with cycle 5 (five patients). Growth factor support was given to six patients (29%) with cycles 3 (two patients), 4 (five patients), 5 (one patient) and 6 (three patients).

### Radioembolization dosimetry and administration

^90^Y resin microsphere dosimetry information is summarized in Table 2. A total of 40 administrations of ^90^Y resin microspheres were performed on study for 21 patients. Nineteen patients (90%) underwent two planned lobar radioembolization treatments with cycles 1 and 2 of TAS-102. One patient developed disease progression within the untreated left liver lobe during cycle 1, which led to biliary obstruction. This patient was not a candidate for repeat radioembolization due to sustained hyperbilirubinemia, and clinical trial participation was discontinued. The other patient developed protracted grade 4 neutropenia and had an anticipated need for growth factor support during cycle 2, which was not allowed per protocol, and thus the treating oncologist elected to discontinue trial participation. A total of 21 right hepatic lobe treatments were performed with median delivered activity of 38.9 mCi (range 27.8–72.3 mCi) that corresponded to median absorbed liver dose (MIRD dosimetry) of 65.6Gy (range 21.5–98.5Gy). A total of 19 left hepatic lobe treatments were performed with median delivered activity of 23.3 mCi (range 13.1–43.4 mCi) that corresponded to median absorbed liver dose of 87.6Gy (range 24.8–140.1Gy). Median lung shunt fraction was 5.2% (range 1.4–17.8%), and median total absorbed lung dose was 4.9Gy (range 1.6–27.5Gy).

**Table 2:** ^90^Y Resin microsphere dosimetry

Parameters	Median (range)
Administered activity, mCi	
Right lobe	38.9 (27.8–72.3)
Left lobe	23.3 (13.1–43.3)
Total	66.3 (34.3–115.6)
Liver volume, ml	
Right lobe	1151 (662–2760)
Left lobe	584 (265–900)
Absorbed liver dose, Gy*	
Right lobe	65.6 (21.5–98.5)
Left lobe	87.6 (24.8–140.1)
Lung shunt fraction, %	5.2 (1.4–17.8)
Absorbed lung dose, Gy*	4.9 (1.6–27.5)

*MIRD model calculations

### Safety

The maximum tolerated dose of TAS-102 was not reached. Maximum FDA-approved dose of 35 mg/m^2^ (maximum 80 mg) was adopted for dose expansion phase (12 patients). AEs are summarized in Table 3. The most common clinical AEs were abdominal pain (16 patients, 76%), nausea (13 patients, 62%), diarrhea (12 patients, 57%), fatigue (11 patients, 52%), vomiting (8 patients, 38%), and anorexia (8 patients, 38%). Common metabolic and laboratory AEs were neutropenia (15 patients, 71%), thrombocytopenia (13 patients, 62%), anemia (11 patients, 52%), AST elevation (14 patients, 67%), hyperbilirubinemia (8 patients, 38%), and hypoalbuminemia (7 patients, 33%). Severe AEs included neutropenia (10 patients), anemia (5 patients, hyperbilirubinemia (2 patients), hypoalbuminemia (2 patients), thrombocytopenia (1 patient), and febrile neutropenia (1 patient). Most common causes of TAS-102 dose delay and reduction were severe neutropenia and hyperbilirubinemia.

**Table 3:** Adverse events

Toxicity / Grade	Grade 1, N (%)	Grade 2, N (%)	Grade 3, N (%)	Grade 4, N (%)
**Clinical**				
Abdominal pain	11 (52)	5 (24)	–	–
Anorexia	6 (29)	2 (10)	–	–
Back pain	5 (24)	–	–	–
Constipation	5 (24)	–	–	–
Diarrhea	9 (43)	2 (10)	1 (5)	–
Dysgeusia	4 (19)	1 (5)	–	–
Emesis	7 (33)	1 (5)	–	–
Fatigue	7 (33)	4 (19)	–	–
Fever	4 (19)	–	–	–
Headache	3 (14)	–	–	–
Insomnia	4 (19)	–	–	–
Nausea	10 (48)	3 (14)	–	–
Night sweats	3 (14)	–	–	–
**Laboratory**				
ALT elevation	8 (38)	1 (5)	–	–
AST elevation	10 (48)	4 (19)	–	–
Bilirubin elevation	3 (14)	3 (14)	2 (10)	–
Hypoalbuminemia	3 (14)	2 (10)	2 (10)	–
Anemia	3 (14)	3 (14)	5 (24)	–
Leukopenia	2 (10)	4 (19)	4 (19)	1 (5)
Neutropenia	2 (10)	3 (14)	7 (33)	3 (14)
Neutropenic fever	–	–	1 (5)	–
Thrombocytopenia	8 (38)	4 (19)	1 (5)	–

There were two patients (10%) who developed clinical signs of liver failure that included jaundice, ascites, and portal hypertension in the absence of radiographic liver disease progression, which were consistent with radioembolization-induced liver disease (REILD). One patient developed REILD symptoms 5 months after starting study therapy and died of liver failure 2 months later. Another patient developed REILD 5 months after initiation of study therapy. This patient survived 30 months after enrollment and did not develop liver tumor recurrence despite not receiving any additional cancer therapy.

Two additional patients (10%) developed delayed-onset liver toxicity > 12 months after initiation of study therapy. One patient who discontinued study therapy due to cytopenias eventually experienced improvement in blood counts and received 24 additional cycles of TAS-102 off study. This patient developed grade 3 hyperbilirubinemia and ascites 15 months after radioembolization. As of the data censor date, this patient was alive 31 months after starting study therapy. The second patient also discontinued TAS-102 due to prolonged neutropenia, which ultimately improved to allow additional off-study treatments with TAS-102, bevacizumab, TARE, capecitabine, and pembrolizumab. This patient developed grade 3 hyperbilirubinemia and ascites 18 months after the first radioembolization procedure (6 months after off-study TARE) and had several hospital admissions for peristomal variceal bleeds that required embolization via transhepatic route. This patient died 36.8 months after starting study therapy.

### Treatment response

**Fig. 2:**
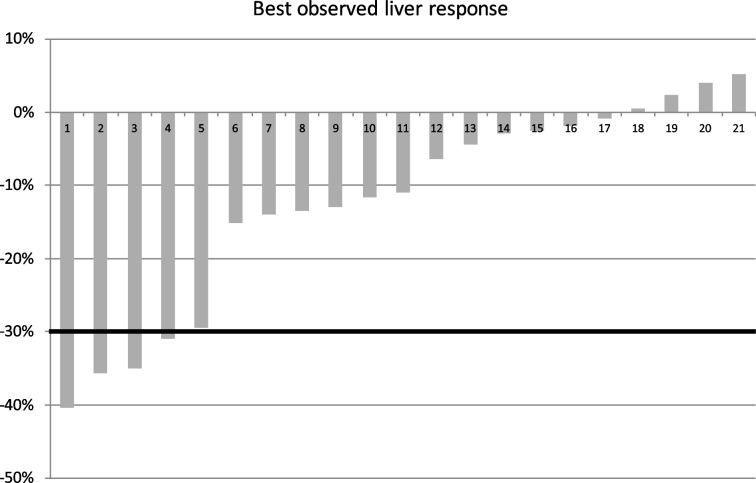
Waterfall plot summarizing maximum observed radiographic responses within the liver by RECIST criteria version 1.1

Data regarding treatment responses are summarized in Table 4. Disease control rate within the treated liver lobes was 100%. Best observed overall radiographic responses (Fig. 2) were PR for 4 patients (19%) and stable disease for 12 patients (57%). Median time to maximum response was 2.1 months (range 1.5–7 months).

**Table 4:** Radiographic and clinical response summary

Parameter	N = 21
Best observed radiographic response, n (%)	
Complete response (CR)	0 (0)
Partial response (PR)	4 (19)
Stable disease (SD)	12 (57)
Progressive disease (PD)	5 (24)
Overall response rate (ORR), n (%)	4 (19)
Disease control rate (DCR), n (%)	16 (76)
Time to maximum response, median (range), months	2.1 (1.5–7)
Hepatic progression, n (%)	11 (52)
Extrahepatic progression, n (%)	13 (62)
Extrahepatic disease site	
Lungs	9
Lymph nodes	6
Peritoneum	5
Ovary	2
Primary tumor	2
PFS, median (range), months	
Overall	3.8 (0.7–21.2)
Hepatic	4.4 (1.5–21.2)
Extrahepatic	3.6 (0.7–18.5)
OS, median (range), months	6.4 (3–36.8)

The overall disease control rate was 76%. Five patients (24%) developed disease progression outside of the treated liver while receiving treatment with TARE and TAS-102 during first two treatment cycles. Four of these patients experienced extrahepatic disease progression based on scans obtained after cycle 2, while one patient developed biliary obstruction that resulted from progressive disease within the untreated liver lobe 1 month after the first lobar TARE procedure and precluded further treatment with TARE and TAS-102.

**Fig. 3:**
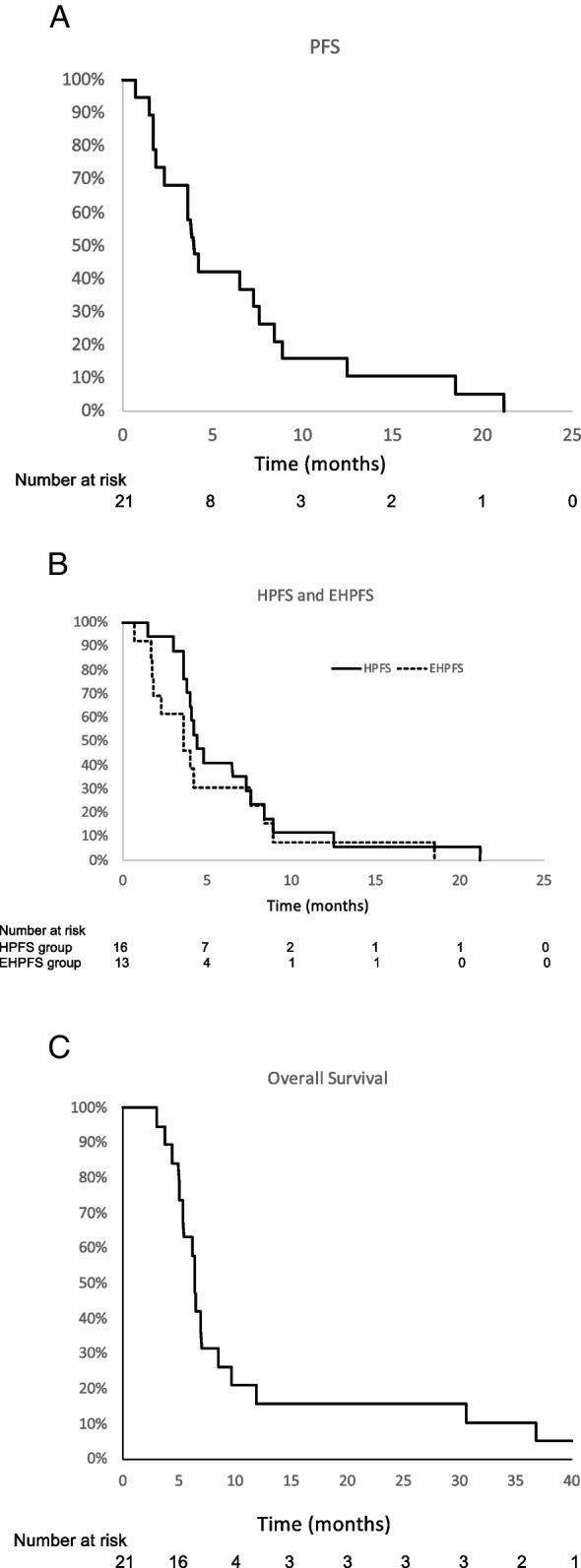
Kaplan-Meier plots of progression free survival **A**, hepatic and extrahepatic progression free survival **B**, and overall survival **C**

A total of 11 patients (52%) demonstrated liver disease progression, 13 patients (62%) developed progression outside the liver (most commonly involving lungs, lymph nodes, and peritoneal cavity), and three patients were taken off study due to clinical deterioration. Two patients discontinued study participation due to persistent severe cytopenias that precluded administration of additional TAS-102. Median overall progression free survival (Fig. 3A) was 3.8 months (range 0.7–21.2 months), while median hepatic PFS was 4.4 months (range 1.5–212 months), and median extrahepatic PFS (Fig. 3B) was 3.6 months (range 0.7–18.5 months). Despite a trend towards longer hepatic PFS within the first 6 months of starting study therapy, the difference between hepatic and extrahepatic PFS was not statistically different (*p* = 0.40). Median and mean overall survival (Fig. 3C) were 6.4 months and 9.4 months, respectively, with the range of 3–36.8 months. There was one patient alive at the time of data censor date (31 months following enrollment).

As of the data censor date four patients (21%) underwent additional anti-cancer therapy following discontinuation of study participation. Treatments included TAS-102 off-study (3 patients, with bevacizumab added for one patient), FOLFOX (1 patient), capecitabine (1 patient), panitumumab with and without irinotecan (1 patient), pembrolizumab (1 patient), radioembolization with ^90^Y resin microspheres (2 patients), and external beam radiation to a solitary lung metastasis and to a gastrohepatic ligament lymph node (1 patient). Of the two patients treated with repeat radioembolization, one of was treated in combination with TAS-102 off study due to severe neutropenia that necessitated growth factor support with cycle 2 of treatment (not permissible per protocol), while another patient was treated with radioembolization 1 year following clinical trial discontinuation due to localized disease progression within the liver.

## Discussion

The current study demonstrated that there were no dose-limiting toxicities when TAS-102 was combined with lobar TARE using ^90^Y resin microspheres. Standard ^90^Y dose based on the commonly employed BSA model was administered. The full dose of TAS-102 recommended for use by the US Food and Drug Administration of 35 mg/m^2^ (up to 80 mg) twice daily on days 1–5 and 8–12 of 28-day cycles was tested in combination with TARE. This dose schedule was slightly modified by omitting the morning doses of TAS-102 on day 3 of cycles 1 and 2 due to patients’ fasting status and administering them on day 13 of cycles 1 and 2. Seventeen of 19 (89%) patients who received a second cycle of TAS-102 and ^90^Y radioembolization did not require TAS-102 dose reduction with cycle 2. Thirteen out of 15 (87%) patients who received a third cycle of TAS-102 were treated with the full dose of the drug. Dose delays and dose reductions were common due to cytopenias, consistent with reports from prior studies [11, 16, 17].

The treatment toxicity profile of TARE was also consistent with prior reports, with AEs including fatigue, abdominal pain, nausea, vomiting, and liver toxicities including hyperbilirubinemia, transaminitis, and hypoalbuminemia [5–8, 10]. Of note, the pattern of radioembolization-induced liver disease consisting of jaundice, ascites, and portal hypertension in the absence of radiographic liver disease progression was observed for two (10%) patients. These two patients ultimately died of liver failure 7 and 30 months after the initiation of study therapy. Two additional patients developed hyperbilirubinemia, ascites, and portal hypertension more than a year following on-study radioembolization, but were able to receive additional potentially hepatotoxic anti-cancer therapies after trial participation was completed and before the onset of liver disease.

None of the patients developed progressive disease within the liver lobes treated with TARE while receiving active treatment with the combination of radioembolization and TAS-102, which underlines the effectiveness of radioembolization for short-term local control of colorectal liver metastases, even in salvage treatment setting. Disease control rate in this study was 76% and was limited by the ability of TAS-102 to control progression outside of the liver lobe(s) treated with TARE. Conversely, DCR has been reported to be 56% for TARE alone [5], suggesting that TAS-102 may have added benefit, including the effect of radiosensitization. One study [10] that evaluated patients with chemotherapy-refractory bilobar mCRC with cross-sectional imaging one month following each lobar TARE found that median duration of response within the untreated liver lobe was 1 month, and that 13 out of 18 patients (72%) with bilobar disease distribution were unable to receive the second planned lobar TARE due to disease progression within the untreated liver lobe. Addition of TAS-102 in the current trial allowed 19 patients (90%) to complete both planned lobar TARE procedures without experiencing disease progression within the lobe that was treated last. There was one additional patient who received second lobar TARE with TAS-102 off study. Repeat treatment with TARE may be feasible and safe in select patients with mCRC who develop liver disease progression in the setting of stable or absent extrahepatic disease [18]. For these patients, addition of TAS-102 to TARE for its radiosensitizer property may be a reasonable consideration.

Median and mean overall survival in this study were 6.4 months and 9.4 months, respectively, with the survival duration exceeding 1 year for 4 patients (19%). Median overall survival in this study was similar to TAS-102 monotherapy (6.7–7.1 months) [11, 17], but was shorter than OS reported for TARE performed in combination with capecitabine (median 8.1 months, mean 15.3 months) [19] or with 5-FU (10 months) [6]. Shorter median OS in this cohort than in studies involving TARE and other fluoropyrimidines may have been due to differences in patient population, with more heavily pretreated patients referred for TARE at our institution than at other centers.

This study has several limitations. Due to the small sample size, additional data are needed to confirm the safety and efficacy profile for TARE in combination with TAS-102. Radioembolization using two-compartment ^90^Y dosimetry approach that may result in enhanced tumor absorbed doses [20–23] than the body surface area dosimetry method should be employed for a future study of TARE in combination with TAS-102. Considering the non-negligible rate of hepatotoxicity in this trial as well as other studies [5–8, 10, 18], two-compartment dosimetry may permit assessment of radiation doses to the healthy liver and may reduce the risk of hepatotoxicity. Safety of whole-liver TARE in combination with TAS-102 could also be evaluated. Inclusion of patients with limited extrahepatic disease may have diminished the ability to detect the clinical benefit of ^90^Y radioembolization. Based upon data published following development of this protocol, TAS-102 is frequently administered in combination with bevacizumab in salvage setting, rather than as a single agent [17]. While the use of bevacizumab is contraindicated around the time of angiography procedures due to increased risk of catheterization-related vascular adverse events, such as arterial dissections [24], treatment with the combination of TAS-102 and bevacizumab may be a better therapeutic strategy after completion of radioembolization treatments [17].

In conclusion, TAS-102 could be safely administered at the full recommended dose of 35 mg/m^2^ (maximum 80 mg) together with TARE (body surface area dosimetry model). However, dose delays and reductions were often necessary due to cytopenias. TARE in combination with TAS-102 resulted in disease control within the targeted liver lobes for all patients. A majority of the patients (62%) developed extrahepatic disease progression after median of 3.6 months, and 52% of the patient had disease progression within the liver after a median of 4.4 months, suggesting that novel systemic therapy options are needed for maintenance of disease response following ^90^Y radioembolization.

## Supplementary Information

**Additional file 1.**

## Data Availability

Deidentified data set was provided as a supplementary file attachment for review.
